# Dysregulated miR34a/diacylglycerol kinase ζ interaction enhances T-cell activation in acquired aplastic anemia

**DOI:** 10.18632/oncotarget.14046

**Published:** 2016-12-20

**Authors:** Yuan-xin Sun, Hui Li, Qi Feng, Xin Li, Ying-yi Yu, Li-wei Zhou, Yan Gao, Guo-sheng Li, Juan Ren, Chun-hong Ma, Cheng-jiang Gao, Jun Peng

**Affiliations:** ^1^ Department of Hematology, Qilu Hospital, Shandong University, Jinan, China; ^2^ Department of Rheumatology, People's Hospital of Bao’an, Shenzhen, China; ^3^ Shandong Provincial Key Laboratory of Immunohematology, Qilu Hospital, Shandong University, Jinan, China; ^4^ Department of Hematology, First Affiliated Hospital, Xi’an Jiaotong University, Xi’an, China; ^5^ Department of Immunology, Shandong University School of Medicine, Jinan, China; ^6^ Key Laboratory of Cardiovascular Remodeling and Function Research, Chinese Ministry of Education and Chinese Ministry of Health, Jinan, China

**Keywords:** aplastic anemia, miR34a, diacylglycerol kinase ζ, T cells

## Abstract

Acquired aplastic anemia is an idiopathic paradigm of human bone marrow failure syndrome, which involves active destruction of hematopoietic stem cells and progenitors by cytotoxic T cells in the bone marrow. Aberrant expression of microRNAs in T cells has been shown to lead to development of certain autoimmune diseases. In the present study, we performed a microarray analysis of miRNA expression in bone marrow CD3^+^ T cells from patients with aplastic anemia and healthy controls. Overexpression of miR34a and underexpression of its target gene diacylglycerol kinase (DGK) ζ in bone marrow mononuclear cells were validated in 41 patients and associated with the severity of aplastic anemia. Further, the level of miR34a was higher in naïve T cells from patients than from controls. The role of miR34a and DGKζ in aplastic anemia was investigated in a murine model of immune-mediated bone marrow failure using miR34a^−/−^ mice. After T-cell receptor stimulation *in vitro*, lymph node T cells from miR34a^−/−^ mice demonstrated reduced activation and proliferation accompanied with a less profound down-regulation of DGKζ expression and decreased ERK phosphorylation compared to those from wild-type C57BL6 control mice. Infusion of 5 × 10^6^ miR34a^−/−^ lymph node T cells into sublethally irradiated CB6F1 recipients led to increased Lin^-^Sca1^+^CD117^+^ cells and less vigorous expansion of CD8^+^ T cells than injection of same number of wild-type lymph node cells. Our study demonstrates that the miR34a/DGKζ dysregulation enhances T-cell activation in aplastic anemia and targeting miR34a may represent a novel molecular therapeutic approach for patients with aplastic anemia.

## INTRODUCTION

Acquired aplastic anemia (AA) is an idiopathic paradigm of human bone marrow failure syndromes, characterized by bone marrow (BM) aplasia and peripheral blood pancytopenia. Patients with AA often present with symptoms of anemia, hemorrhage, and frequent infection. The pathophysiology of AA remains unclear, but in most cases AA behaves as an immune-mediated disease in which the cytotoxic T lymphocytes destroy hematopoietic stem/progenitor cells in the bone marrow [1–3]. Up-regulation of CD3 gene expression in AA patients suggests that T cells may be receiving sustained stimulation signaling that leads to inappropriate T cell activation [4]. The responsiveness of AA patients to immunosuppressive therapy with anti-thymocyte globulin and cyclosporine strongly support the underlying immune pathogenesis [5, 6]. A large amount of laboratory data also provided evidence for the pivotal role of the immune system in the disease pathophysiology [7]. Nevertheless, the existing data does not completely explain the mechanism of effector T cells involved in AA.

MicroRNAs (miRNAs) are small conserved non-coding RNAs that negatively modulate the expression of complementary genes. Over 60% of the human coding genes are estimated to be under miRNA control, and a single microRNA can potentially regulate hundreds of target genes [8–11]. miRNAs have been shown to be important regulators of immune homeostasis and their aberrant expression was found in some autoimmune diseases, such as miR155 and miR146a in rheumatoid arthritis, and miR145 and miR224 in lupus erythematosus [12–14]. Hosokawa et al. reported that expression of four miRNAs (miR126-3p, miR145-5p, miR199a-5p, and miR223-3p) were decreased in peripheral blood T cells of AA patients [15]. However, information regarding miRNA regulation in BM T cells of AA patients is lacking.

Bone marrow microenvironment is the place hematopoietic stem cells are attacked by immune cells in AA. Previously, we demonstrated that elevated expression of CX3C chemokine receptor 1 mediated recruitment of T cells into the bone marrow of patients with AA [16]. Maciejewskiet al. proposed that BM phenotyping was more sensitive than peripheral blood analysis for detecting the abnormal cellular immune response in AA, since the percentage of activated CD8^+^ cells were more easily detected in BM than in peripheral blood from AA patients [17]. Thus, in the present study, we performed a microarray analysis of the miRNA expression patterns in BM CD3^+^ T cells of AA patients and healthy controls to screen for miRNAs differentially expressed in AA. The miRNAs level between AA and controls were verified by quantitative real time-polymerase chain reaction (RT-PCR) in 41 patients and 20 healthy individuals. Notably, miR34a expression was higher in all the 41 patients than in healthy controls.

miR34a has been identified as a direct transcriptional target of p53 [18, 19]. Similar to the p53 tumor suppressor gene, miR34a inhibits cell proliferation and induces apoptosis. However, the function of miR34a in hematopoiesis and the immune response is largely unknown. Of note, Shin et al. demonstrated that miR34a directly targets diacylglycerol kinase (DGK) ζ via its seed matches in both coding region and 3’ untranslated region by the way of luciferase reporter assay [20]. DGKζ is a member of the DGK family that catalyzes conversion of diacylglycerol (DAG), a crucial second messenger of receptor-mediated signaling in T cell activation, to phosphatidic acid. In T cells DAG is required for activation of diverse downstream signaling cascades, including the Ras-extracellular signal regulated kinase (Ras-ERK) and nuclear factor-κB pathways. Through phosphorylation of DAG, DGKζ terminates or inhibits downstream signaling, thus controlling DAG in T cell activation. The role of DGKζ in T cell receptor (TCR) signaling has been investigated in both cell lines and murine models [21–23]. However, whether DGKζ participates in the pathogenesis of AA was previously unknown.

Therefore, in the present study, we first identified and characterized the overexpressed miR34a and its corresponding target gene DGKζ in T cells of AA patients. We then generated a murine model of bone marrow failure and explored the possible roles of miR34a and DGKζ in T cell activation in AA. Our findings indicate for the first time that dysregulated miR34a/DGKζ interaction plays a critical role in the abnormal T cell immunity in AA and provide a base for developing new therapeutic target for AA.

## RESULTS

### miRNA expression profiles in BM T cells from AA patients and healthy controls

**Figure 1:**
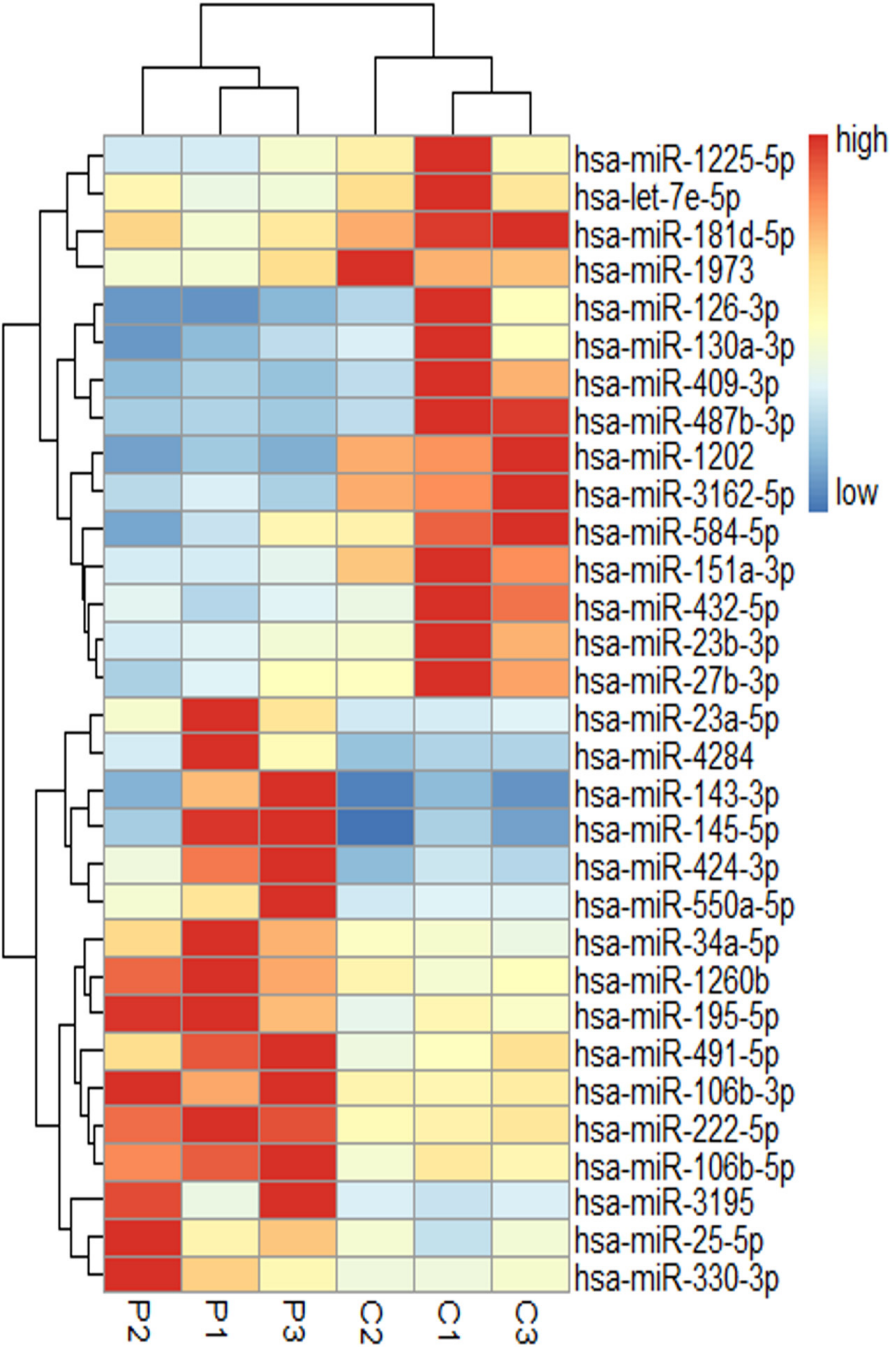
miRNA expression profiles in BM T cells of SAA patients and controls P indicates SAA patients (*n* = 3), C is controls (*n* = 3). miRNA are in rows, samples in columns. For each miRNA, red represents an expression value higher than the average expression across all samples, blue represents an expression value below average.

Among the 1105 hsa-miRNAs, we identified 31 miRNAs with significantly differential expression between three SAA patients and three healthy individuals. Of these, 16 were overexpressed and 15 were underexpressed in disease *vs* health. Figure 1 depicts a heat map illustrating the differentially expressed miRNAs in either direction. Differential expression analysis between the two groups was analyzed based on the criteria of fold-change ≥ 1.5 and *P value* < 0.05. The microarray data described in this report have been deposited in NCBIs Gene Expression Omnibus ( http://www.ncbi.nlm.nih.gov/geo/) and are accessible through Gene Expression Omnibus Series accession number GSE82095.

### miR34a overexpression in bone marrow mononuclear cells (BMMCs) is associated with the severity of AA

**Figure 2:**
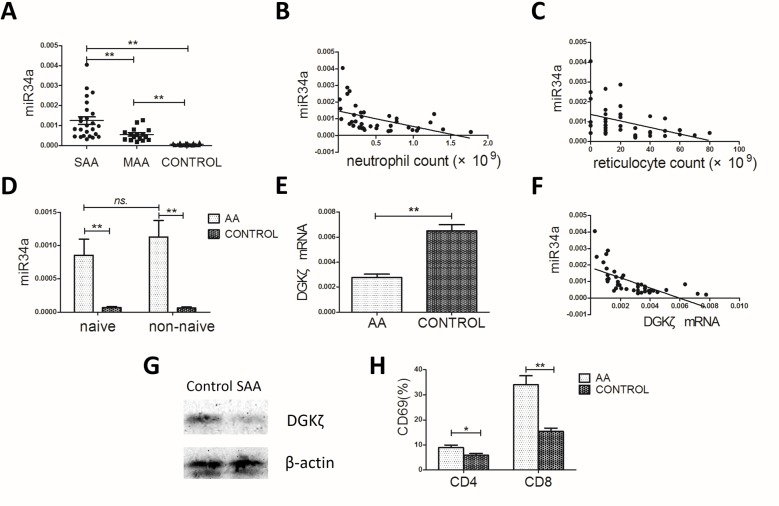
miR34a, DGKζ and CD69 expression in the AA patients and controls (**A**) miR34a expression in AA patients (*n* = 41) was much higher than in controls (*n* = 20), while in SAA group (*n* = 25) it's much higher than in MAA group (*n* = 16) (one-way ANOVA, with LSD post-test). (**B, C**) Correlations between miR34a level and peripheral blood neutrophil count or reticulocyte count in the 41 AA patients (Spearman's test). (**D**) miR34a expression in naïve T and non-naïve T cells from SAA patients (*n* = 8) and controls (*n* = 8). (**E**) DGKζ mRNA level in BMMCs (Student's *t*-tests). (**F**) Negative correlation between DGKζ mRNA level and miR34a level in AA patients (Spearman's test). (**G**) The representative data of DGKζ protein expression in BMMCs from SAA patients (*n* = 8) and controls (*n* = 8). (**H**) The proportion of surface CD69^+^ cells among CD4^+^ and CD8^+^ BMMC in AA patients and controls (Student's *t*-tests). Mean ± SEM. *P < 0.05; **P < 0.01.

The differentially expressed miRNAs were examined further by RT-PCR in BMMCs from 41 AA patients and 20 healthy controls. The expression of miR34a in the SAA and MAA groups were both significantly higher than in healthy controls (12.6 ± 9.44 × 10^–4^ *vs* 5.63 ± 3.17 × 10^–4^ *vs* 0.74 ± 0.48 × 10^–4^; *P* < 0.001 for the two comparisons; Figure 2A). In addition, miR34a expression was associated with AA severity, higher in the SAA group than in the MAA group (*P* = 0.002; Figure 2A). Negative correlations between miR34a levels and peripheral blood neutrophil or reticulocyte counts were observed in AA patients (r = –0.472, *P* = 0.002; r = –0.475, *P* = 0.002; Figure 2B–2C). We found no significant correlations with peripheral red blood cell count, lymphocyte count, or platelet count (Supplementary Figure S1). Besides, the level of miR34a in naïve T cells and non-naïve T cells from AA patients was both much higher than from healthy controls (naïve T cells: 9.66 ± 6.81 × 10^–4^ *vs* 0.69 ± 0.52 × 10^–4^, *P* = 0.007; non-naïve T cells: 11.32 ± 7.01 × 10^–4^ *vs* 0.63 ± 0.44 × 10^–4^, *P* = 0.003; Figure 2D) and no significance were observed between naïve T cells and non-naïve T cells.

### DGKζ is downstream target gene of miR34a in BMMCs from AA patients

Referring to previous studies [24, 25] and using a target prediction and validation program, miRWalk 2.0 [26], we chose 7 potential target genes of miR34a to examine further in AA patients and healthy individuals. These genes included Kruppel-like factor 4 (KLF4), lymphoid enhancer binding factor 1 (LEF1), SPI-1 proto-oncogene (SPI1), nuclear receptor subfamily 4 group A member 2 (NR4A2), sirtuin 1 (SIRT1), cyclin-dependent kinase 6 (CDK6), and DGKζ. Expression of the first 6 potential target genes was not different between the two groups (Supplementary Figure S2). However, DGKζ mRNA levels were significantly reduced in AA patients compared to controls (0.00277 ± 0.00173 *vs* 0.00649 ± 0.00222; *P* < 0.001; Figure 2E) and negatively correlated with miR34a levels (r = –0.662, *P* < 0.001; Figure 2F). Immunoblot analysis confirmed the low expression of DGKζ in the BMMCs of AA patients (Figure 2G).

DGKζ has been demonstrated to be a direct target of miR34a and to play an important role in T cell activation [20]. Thus, we measured the T cell activation marker CD69 on BMMCs by flow cytometry. The proportion of surface CD69^+^ cells among CD4^+^ or CD8^+^ BMMCs in AA patients was significantly higher than in controls (CD4^+^ cells: 8.90 ± 3.28 *vs* 5.59 ± 2.05, *P* = 0.027; CD8^+^ cells: 34.09 ± 11.10 *vs* 15.34 ± 4.3, *P* < 0.001, respectively; Figure 2H). Taken together, the altered expression of the miR34a and its predicted target DGKζ in BMMCs, especially the high miR34a level at the naïve T cell stage before T cell differentiation and activation in AA, suggest an active role of the miR34a-DGKζ-T cell activation signaling in the pathogenesis of AA.

### miR34a down-regulation decreases activation of T cells from AA patients

**Figure 3:**
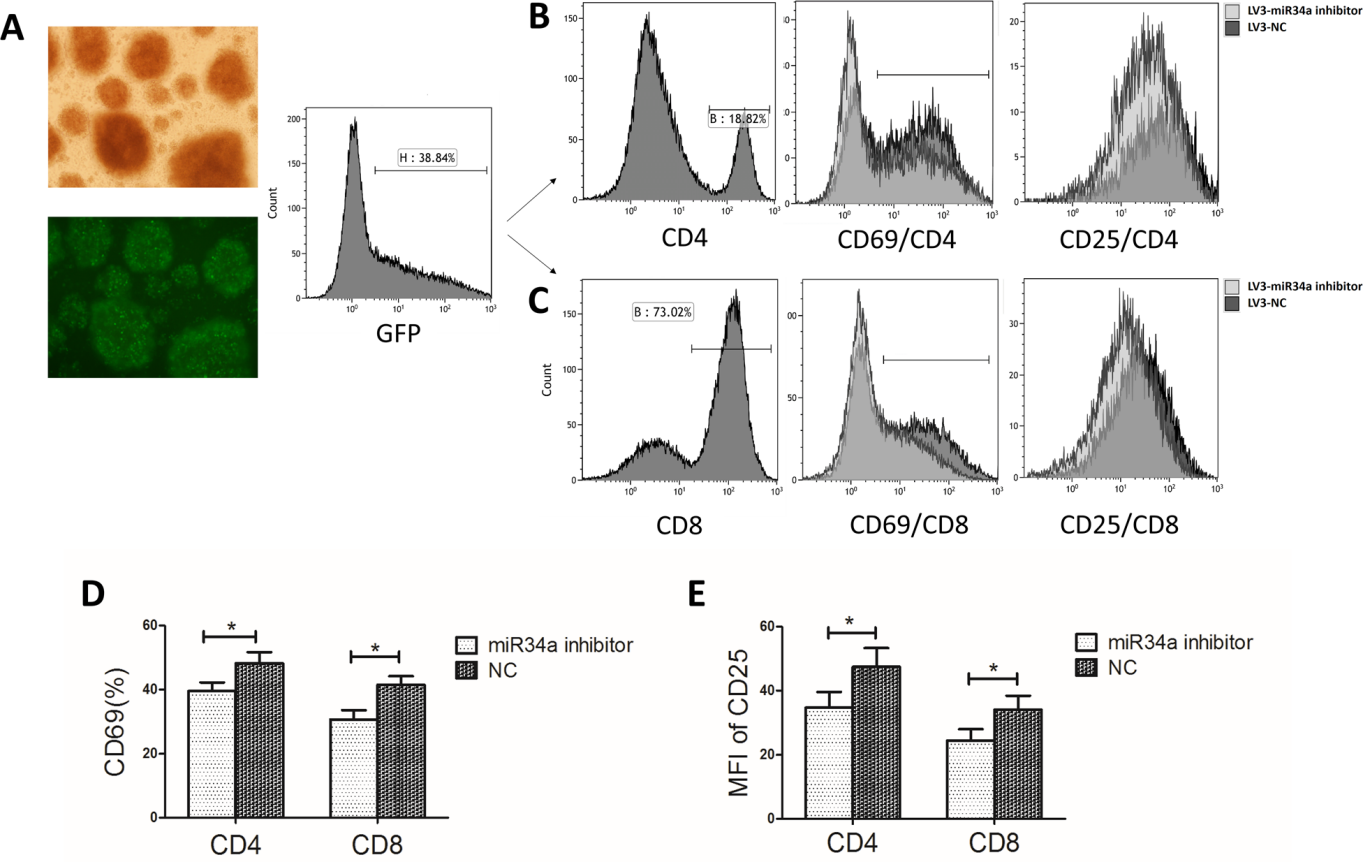
T cell activation after transfection with miR34a inhibitor into BMMCs from ten randomly selected AA patients (**A**) BMMCs from one AA patient transfected with LV3-miR34a inhibitor and LV3-NC. GFP^+^ cells were shown in the left panel (original magnification 100 ×). The histogram of flow cytometry showed the transfection rate. (**B–E**) CD4^+^ and CD8^+^ cells were gated from the GFP^+^ cells. The CD69 and CD25 level on CD4^+^ or CD8^+^ cells transfected with LV3-miR34a inhibitor and LV3-NC were presented as the four merged histograms (B, C) and means with SEMs (D, E: Paired sample *t-test*).*P < 0.05.

To explore the effect of miR34a on T cell activation and growth, BMMCs from AA patients were transfected with lentivirus carrying miR34a inhibitor sequences (LV3-miR34a inhibitor) or non-specific sequences (LV3-NC). RT-PCR was used to confirm reduced miR34a expression and inversed expression of miR34a and DGKζ in the miR34a inhibitor-transfected group. Five days after transfection, percentage of green fluorescent protein (GFP)-expressing cells in the two groups was similar, approximately 35% (Figure 3A). The CD4^+^ and CD8^+^ cells were gated among the GFP-expressing cells by flow cytometry. The CD4^+^ or CD8^+^ cells transfected with LV3-miR34a inhibitor expressed less CD69 and CD25, the T cell activation marker, than those transfected with LV3-NC, as indicated by percentage of CD69^+^ cells and the mean fluorescence intensity of CD25 (Figure 3B–3E). These data showed miR34a-down-regulated T cells from AA patients expressed lower CD69 and CD25 with a weakly activated state.

### Lymphohematopoietic cellularity in miR34a-deficient mice

The effect of miR34a deficiency on mouse lymphohematopoiesis was analyzed by comparing the cellular composition in blood, BM, spleen, and lymph node between miR34a^−/−^ and wild-type mice. In peripheral blood, miR34a^−/−^ mice showed no significant change in white blood cell, red blood cell, and platelet counts compared to wild-type mice. In BM, the total number of BM cells and BM Lin^-^Sca1^+^CD117^+^ (LSK) hematopoietic stem and progenitor cells [27] were similar between miR34a^−/−^ and wild-type mice. We specifically analyzed the proportions of CD4^+^ and CD8^+^ T cells from peripheral blood, BM, lymph nodes, and spleen. Deletion of miR34a resulted in a mild decrease in CD4^+^ cells in peripheral blood and BM, and had no major effect on other lymphohematopoietic cell types (Table 1).

**Table 1:** Cellular composition in wild-type and miR34a^−/−^ mice

Measurements	WT	miR34a^−/−^	*P*
N(M+F)	5 + 5 = 10	6 + 6 = 12	
**Peripheral blood**			
WBC, ×10^9^/L	12.6 ± 3.90	11.6 ± 2.47	0.463
RBC, ×10^12^/L	10.1 ± 1.59	9.62 ± 1.24	0.453
PLT, ×10^9^/L	1211 ± 214.8	1236 ± 217.0	0.783
CD4, %	14.4 ± 2.09	11.4 ± 1.78	0.018
CD8, %	8.40 ± 1.20	6.83 ± 2.03	0.112
**Bone marrow**			
BM cells, ×10^7^	21.86 ± 1.48	21.18 ± 1.16	0.493
LSK, ×10^5^	8.34 ± 1.52	10.81 ± 1.78	0.088
CD4, %	3.52 ± 0.50	2.10 ± 0.26	0.002
CD8, %	3.53 ± 1.44	2.30 ± 0.71	0.178
**Spleen**			
CD4, %	23 ± 3.67	19.3 ± 1.12	0.063
CD8, %	12.78 ± 3.43	12.36 ± 2.05	0.820
**Lymph nodes**			
CD4, %	36.33 ± 3.41	34.45 ± 9.13	0.618
CD8, %	23.45 ± 3.72	28.29 ± 4.75	0.069

Mean ± SD after *t* test. BM, bone marrow; RBC, red blood cells; WBC, white blood cells; PLT, platelets; LSK, Lin^-^Sca1^+^CD117^+^ cells. Total BM cells were calculated assuming bilateral tibias, ilia, and unilateral femur contain 25% of total marrow cells.

### miR34a deficiency attenuates T cell activation and DGKζ down-regulation in response to TCR stimulation

**Figure 4:**
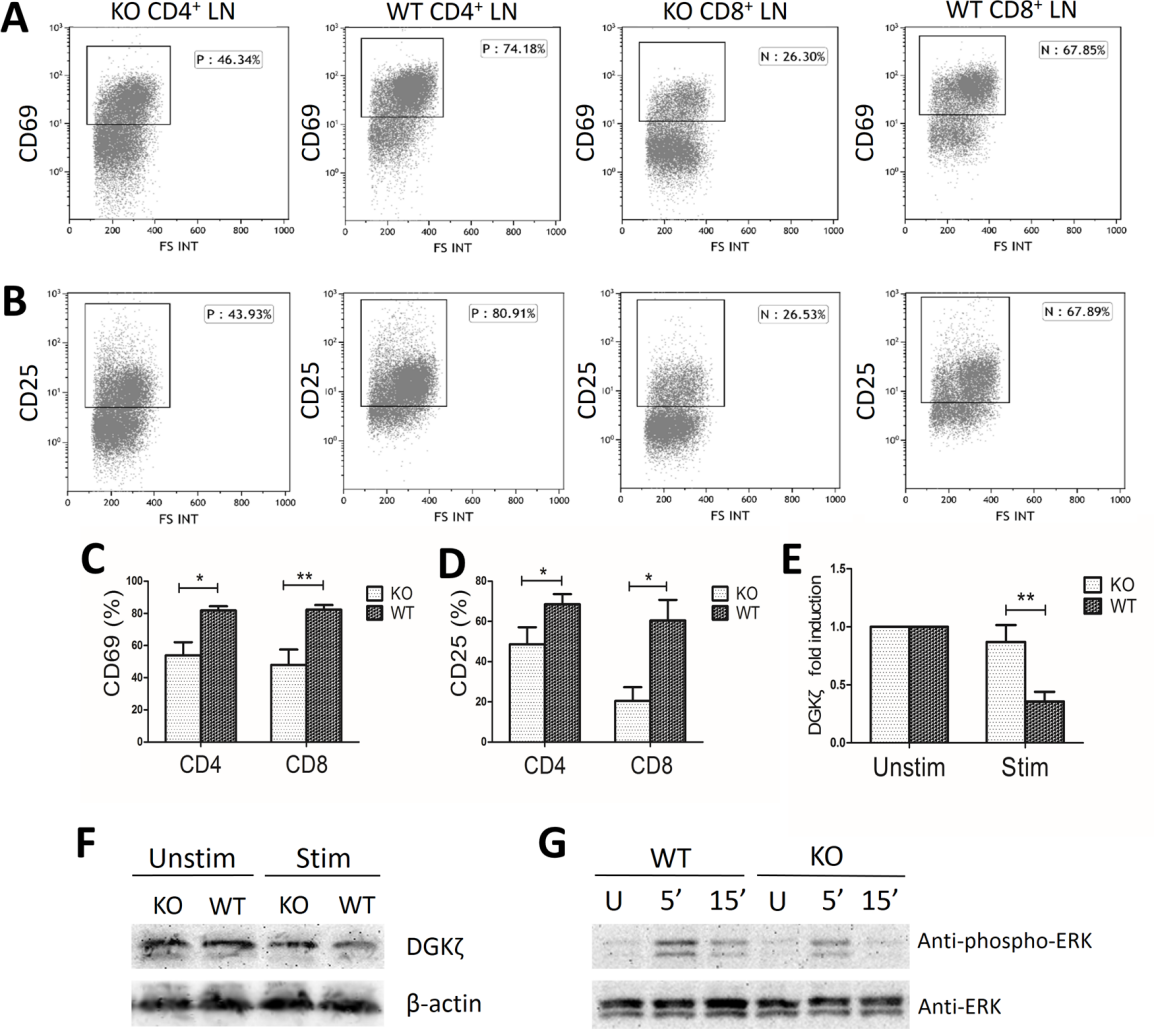
Decreased activation markers and ERK phosphorylation in miR34a-deficient T cells WT, wild type; KO, miR34a-knockout; LN, lymph node. (**A, C**) Percentage of CD69^+^ cells and (**B, D**) CD25^+^ cells in LN CD4^+^ and CD8^+^ cells extracted from wild-type (*n* = 6) and KO (*n* = 6) mice after stimulation with anti-CD3 and anti-CD28 mAbs for 18h. (C and D: Mean ± SEM.) (**E**) Fold induction of DGKζ mRNA levels in KO (*n* = 6) and wild-type (*n* = 6) LN cells reflects the mRNA levels (2^-(CTtarget-CTcontrol)^) of stimulated cells divided by the mRNA levels of unstimulated cells (Mean ± SEM). (**F**) Representative data of decreased DGKζ protein expression in KO and wild-type LN cells after stimulation in three experiments. LN cells for immunoblot were extracted from nine WT and nine KO mice for one experiment. (**G**) Decreased ERK activation in miR34a-deficient LN cells after TCR engagement. LN cells from WT and KO mice were left unstimulated (U) or were stimulated with mAbs to CD3 and CD28 in 5 min and 15 min. Total ERK were determined as loading control. Data are representative of three experiments. LN cells for immunoblot were extracted from nine WT and nine KO mice for one experiment. *P* values were obtained by Student's *t*-tests. *P < 0.05; **P < 0.01.

To study the effect of miR34a deficiency on T cell activation, we stimulated lymph node (LN)cells and splenocytes from miR34a-deficient and wild-type mice with anti-CD3 and anti-CD28 mAbs for 18 h. CD4^+^ and CD8^+^ LN cells from miR34a^−/−^ mice expressed lower levels of CD69 and CD25 than those from wild-type mice (CD69: 53.99 ± 19.86% *vs* 81.90 ± 6.27%, *P* = 0.017 for CD4^+^ cells, and 47.98 ± 26.97% *vs* 82.29 ± 8.22%, *P* = 0.008 for CD8^+^ cells, respectively; CD25: 48.63 ± 16.93% *vs* 68.5 ± 10.08%, *P* = 0.043 for CD4^+^ cells, and 20.44 ± 15.29% *vs* 60.5 ± 22.83%, *P* = 0.012 for CD8^+^ cells, respectively; Figure 4A–4D). The results were qualitatively similar but less notable in the CD4^+^ and CD8^+^ splenocytes.

In the wells without stimulation, DGKζ mRNA levels were not significantly different between miR34a^−/−^ and wild-type cells. In the wells with stimulation, DGKζ expression declined in both groups. In miR34a^−/−^ LN cells, DGKζ mRNA levels decreased to a lesser extent than in wild-type cells (0.869 ± 0.254 fold *vs* 0.357 ± 0.184 fold, *P* = 0.008, Figure 4E). The protein level of DGKζ was consistent with the declined mRNA level after stimulation in the two groups (Figure 4F).

It has been demonstrated that DGKζ negatively regulates TCR signaling by selectively interfering with the Ras-ERK pathway [21, 28], while miR34a can negatively control DGKζ expression during T cell activation. Therefore, we assayed activated ERK to investigate the effect of miR34a deficiency on TCR-induced Ras-ERK activation and found decreased ERK phosphorylation in miR34a-deficient LN T cells compared with that of wild-type LN T cells after TCR ligation (Figure 4G).

Collectively, these data indicate that after TCR stimulation miR34a deficiency induces a less profound down-regulation of DGKζ expression, suppresses TCR-mediated ERK activation and decreases the expression of T cell activation marker CD69 and CD25.

### miR34a-deficient T cells are hypoproliferative

**Figure 5:**
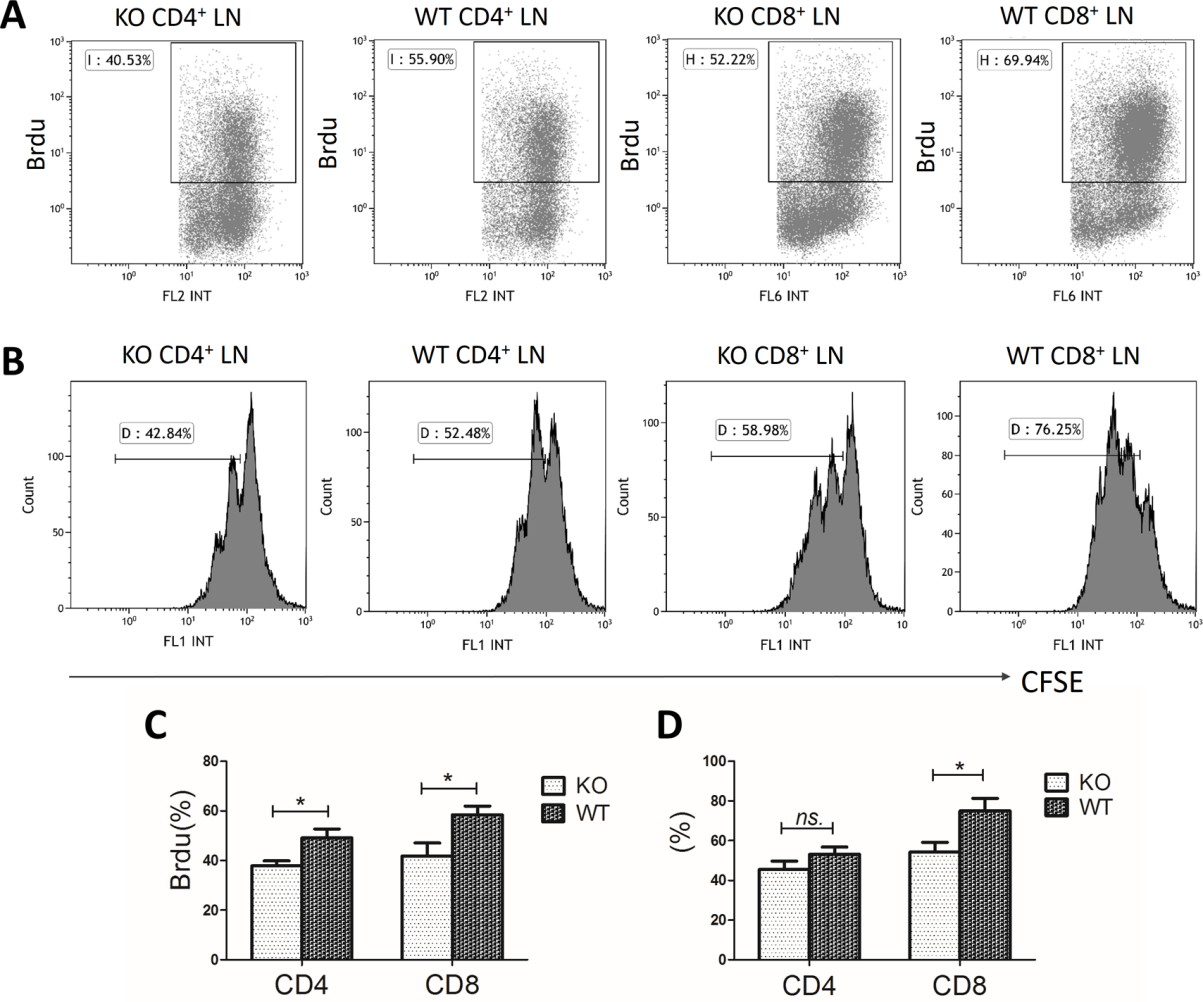
Hypoproliferation of miR34a-deficient T cells WT, wild type; KO, miR34a- knockout; LN, lymph node. (**A, C**) Brdu incorporation rate in wild-type (*n* = 6) and KO (*n* = 6) LN cells after stimulation with anti-CD3 and anti-CD28 mAbs for 72 h. (**B**) Representative histograms of cell division by CFSE labeling with the same treatment as above. (**D**) Percentage of the divided cells. *P* values were obtained by Student's *t*-tests. Mean ± SEM. *P < 0.05; ns, no significance.

Next, we assessed the effect of miR34a deficiency on T cell proliferation. miR34a-deficient CD4^+^ and CD8^+^ LN T cells showed reduced 5-Bromo-2-deoxyuridine (Brdu) incorporation compared with that of wild-type cells after stimulation with anti-CD3 and anti-CD28 mAbs (CD4^+^ cells: 37.8 ± 4.88% *vs* 49.2 ± 8.62%, *P* = 0.019; CD8^+^ cells: 41.9 ± 12.7% *vs* 58.5 ± 8.31%, *P* = 0.023; Figure 5A, 5C).

We also labeled T cells with carboxyfluorescein diacetate succinimidyl ester (CFSE) to trace cell division. CD8^+^ miR34a-deficient T cells divided more slowly than wild-type T cells (54.3 ± 9.61% *vs* 75.2 ± 10.4%, *P* = 0.039; Figure 5B, 5D). No significant difference was observed in CD4^+^ cells although the tendency was similar to that of CD8^+^ cells (45.6 ± 8.53% *vs* 53.1 ± 7.29%, *P* > 0.05; Figure 5B, 5D).

### miR34a deletion impairs T cell function in murine model of BM failure

**Figure 6:**
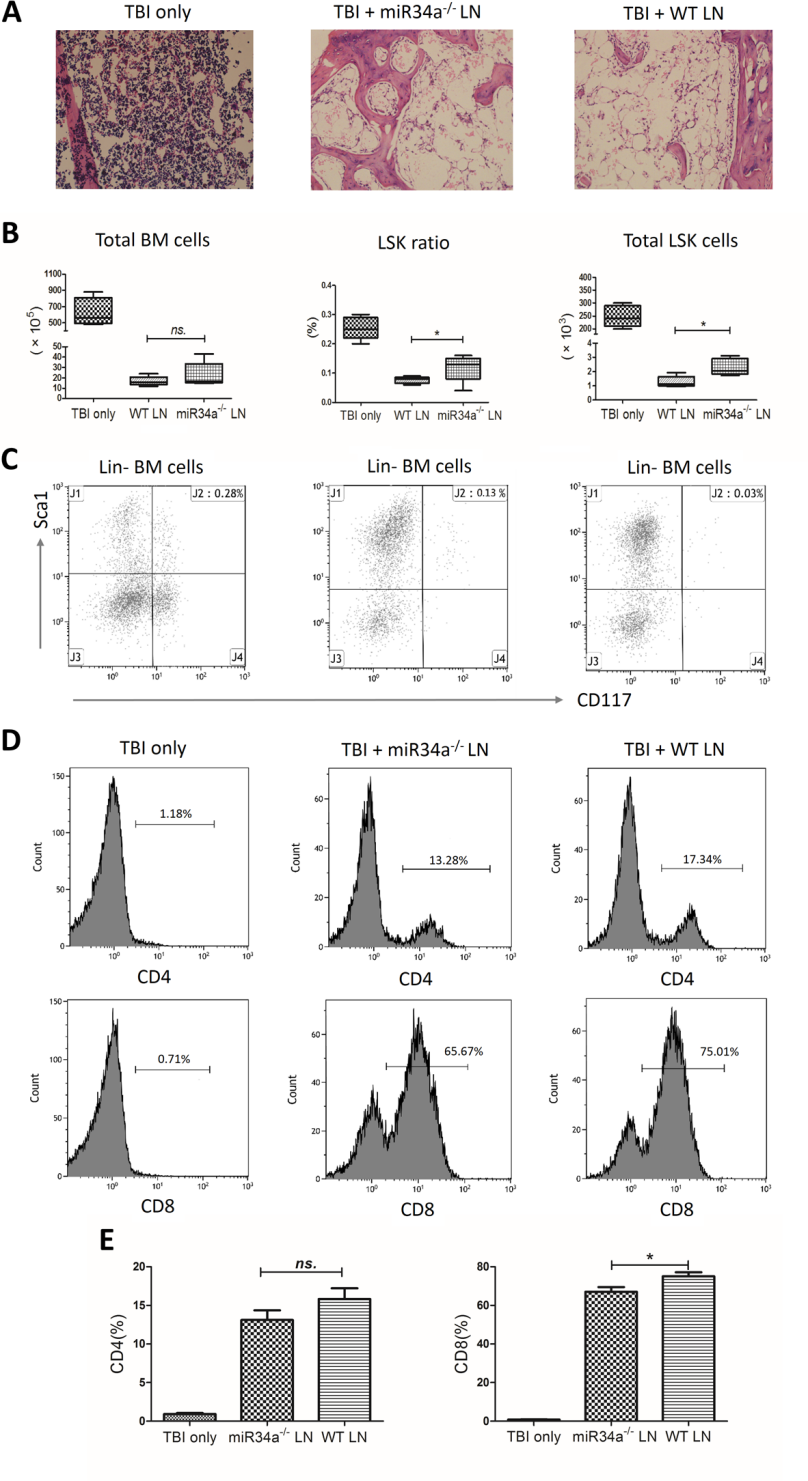
Bone marrow failure model mediated by infusion of wild-type or miR34a−/− lymph node cells Lymph node (LN) cells from wild-type or miR34a^−/−^ donors were used as effectors to induce BM failure in sublethally irradiated (5 Gy) CB6F1 recipient mice. F1 mice were treated with total body irradiation (TBI) only (*n* = 8), TBI with wild-type (WT) LN cells (*n* = 12), or TBI with miR34a^−/−^ LN cells (*n* = 12). Total BM cells were calculated assuming bilateral tibias, ilia, and unilateral femur contain 25% of total marrow cells. (**A**) Representative hematoxylin and eosin femur section (original magnification 200 ×). The three groups were compared in total BM cells, LSK cell percentage, and total LSK cells shown as medians with ranges (**B**) and representative scatterplots (**C**). T cell expansions in the BM are shown as representative histograms (**D**) and means with SEMs (**E**). Infused wild-type LN cells expanded more vigorously than miR34a^−/−^ LN cells. Data are representative of three experiments. *P < 0.05; ns, no significance.

Infusion of wild-type LN cells into sublethally irradiated, MHC-mismatched F1 recipients produced more severe BM hypoplasia compared to infusion of the same number of miR34a-deficient LN cells (Figure 6). The number of total BM karyocytes was reduced in the two infusion groups compared to the total body irradiation (TBI) only group, while no significant difference was found between the two infusion groups (*P* > 0.05; Figure 6A–6B). We also measured the hematopoietic stem and progenitor cells using the previously defined LSK markers, the destruction of which is a characteristic feature of BM failure. Infusion of wild-type LN cells resulted a more significant decrease in the percentage and the total number of LSK cells compared to mice that received miR34a-deficient LN cells (0.076 ± 0.011% *vs* 0.118 ± 0.046%, *P* = 0.043; 1.28 ± 0.38 × 10^3^ *vs* 2.3 ± 0.58 × 10^3^, *P* = 0.011; Figure 6B–6C). In both infusion groups, we observed a decline in white blood cell counts on day 3, platelet counts on day 7, and pancytopenia on day 12 (Supplementary Table S3).

Furthermore, infused wild-type LN cells expanded more vigorously in F1 recipient BMs than miR34a-deficient LN cells. The percentages of CD8^+^ T cells in recipient BM after TBI + wild-type LN cell infusion and TBI + miR34a-deficient LN cell infusion were 75.08 ± 4.65% and 67.03 ± 5.47%, respectively (*P* = 0.037; Figure 6D, 6E). No significant difference was found for CD4^+^ T cells between the two LN cell infusion groups. Collectively, these data indicate that miR34a deficiency impairs the ability of T cells to proliferate and protects against BM failure in an MHC-mismatched recipient environment.

## DISCUSSION

Extensive clinical and laboratory research suggests that acquired AA is a specific autoimmune disease for the aberrant T cell immune homeostasis. Our initial GeneChip assays of BM T cells identified 31 miRNAs with significantly differential expression between three SAA patients and three healthy individuals. In accordance with the results of peripheral blood T cells in Hosokawa's study [15], miR126 was observed to be down-regulated in our microarray analysis, however, its expression was at great variance and the difference between the 41 AA patients and 20 healthy controls was not significant, as analyzed by RT-PCR. The discrepancies might be due to the different miRNA expression in T cells between BM and peripheral blood, or due to the variation in miRNA profiles between different ethnic groups. miR34a was confirmed by RT-PCR to be overexpressed consistently in BMMCs of all the 41 patients. Besides, miR34a expression was higher in SAA than in MAA patients, also negatively correlated with peripheral blood neutrophil and reticulocyte counts in AA patients. These observations indicate that miR34a expression is closely related to AA. Notably, the early change of overexpressed miR34a in naïve T cells from AA patients further suggests that miR34a might contribute to the initiating events resulting in AA.

As an important downstream effector of p53, miR34a is a pro-apoptotic and growth-suppressive miRNA and often acts to mediate repression of many kinds of tumors [29]. In some hematological malignancies, miR34a is dysregulated and plays a role in the pathogenesis by repressing its target genes, such as SIRT1 and FOXP1 [24, 30]. Unlike miR155 and miR146, which have been reported to be aberrantly expressed in many autoimmune diseases [31], little has been reported regarding miR34a. Agnieszka*et al*. detected significantly higher levels of serum IFN-γ and miR34a in peripheral blood mononuclear cells from patients with pulmonary sarcoidosis [32]. miR34a was up-regulated in melanoma cells stimulated with IFN-γ, the type 1 cytokine associated with autoimmune diseases and produced mostly by activated T cells [33, 34]. AA was considered an autoimmune disease in which BM destruction is mediated by effector cells, especially cytotoxic lymphocytes and type 1 cytokines. In present study, we transfected miR34a inhibitor lentivirus into T cells from AA patients. CD69 and CD25 expression on miR34a-inhibited T cells was lower than on control cells. Given this finding, miR34a up-regulation in T cells might partly explain the increased induction and activated state of T cells in AA.

DAG, a second messenger generated by phospholipase Cγ1 activity upon TCR engagement, triggers several signaling cascades that play important roles in T cell development and function. DGKζ is highly expressed in T cells and is a type of DGK that catalyzes the phosphorylation of DAG into phosphatidic acid. DGKζ acts as a breaking mechanism that terminates DAG-mediated signals and negatively regulates T cell activation [28]. Moreover, miR34a has been proved to enhance T cell activation by targeting DGKζ in Jurkat cells [20]. Here, we found that DGKζ mRNA expression in AA was reduced and negatively correlated with miR34a level. No significant differences were found in the mRNA levels of other potential or confirmed target genes. Furthermore, miR34a deficiency in murine LN cells attenuated the decrease in DGKζ expression after stimulation. Together, these results indicate DGKζ may be the working target gene of miR34a in T cells of AA patients. The elevated miR34a level represses DGKζ expression in BM T cells of AA. Upon TCR stimulation DAG was generated from phospholipase Cγ1 and phosphorylated less by the decreased DGKζ, and resulted in the strengthened activation of downstream signaling pathways such as Ras-ERK, ultimately leading to enhanced T cell activation. CD69 and CD25 are activation markers up-regulated in an ERK-mediated manner after TCR ligation [35, 36]. Upon stimulation with anti-CD3 and anti-CD28 mAbs *in vitro*, CD4^+^ and CD8^+^ LN cells from miR34^−/−^ mice expressed much lower levels of CD69 and CD25 and proliferated less vigorously accompanied with reduced ERK phosphorylation than wild-type cells. Consistent with this result, stimulation of Jurkat T cells transduced with miR34a increased CD69 expression and ERK signaling [20].

In our murine model for LN cell-induced BM failure, we found that the miR34a-deficient LN cell infusion weakened CD8 cell expansion, increased the number of total BM hematopoietic stem and progenitor cells, and attenuated the severity of BM hypoplasia. In the model, no significant difference was found in CD4^+^ cell expansion. It is well known that a single microRNA can potentially regulate hundreds of proteins while being regulated by many genes [10]. Therefore, we speculate that the pro-apoptotic and growth-suppressive role of miR34a connected with the p53 network may be involved and have an opposite effect with DGKζ in the BM failure model.

In the current study, we provide direct evidence of the regulatory roles of miR34a and DGKζ in AA patients and in a murine model with miR34a^−/−^ LN cell-mediated BM failure. In AA, high miR34a expression decreased DGKζ level and enhanced T cell activation and proliferation. Targeting miR34a may represent a novel molecular therapeutic approach for patients with AA.

## MATERIALS AND METHODS

### Ethics statement

Investigation has been conducted in accordance with the ethical standards and the national and international guidelines. The clinical study was approved by the Medical Ethics Committee of Qilu Hospital, Shandong University. Written informed consents were obtained from all patients and controls in accordance with the Helsinki Declaration. All animal study protocols were approved by the Animal Ethics Committee of Shandong University School of Medicine.

### Patients and controls

Forty-nine AA patients, 33 with severe AA (SAA) and 16 with moderate AA (MAA), were evaluated at the Department of Hematology, Qilu Hospital, Shandong University, from March 2012 to September 2016. The diagnosis and severity evaluation of AA was established according to the criteria of Camitta *et al*. [37]. All patients were newly diagnosed and without any specific treatment prior to sampling. Twenty eight healthy individuals served as the control group. The clinical characteristics of AA patients and healthy controls, including age, sex, clinical subtypes, neutrophil, platelet, and reticulocyte counts were summarized in Supplementary Table S1.

### Mice

Inbred C57BL/6 (B6, *H2*^b/b^) mice were obtained from the Center for New Drug Evaluation of Shandong University, hybrid CB6F1 (F1, *H2*^b/d^) mice from Vital River Laboratory Animal Technology Co. Ltd (Beijing, China), and induced mutant B6 (Cg)-miR34a^tm1Lhe/J^ (miR34a^−/−^) mice from Jackson Laboratory (Bar Harbor, Maine, USA). All animal experiments were carried out in accordance with the National Institutes of Health guide for the care and use of laboratory animals.

### Affymetrix GeneChip miRNA assay

CD3^+^ cells in bone marrow mononuclear cells (BMMCs) were positively selected from three SAA patients and three healthy controls using an immunomagnetic activated cell sorting system (Miltenyi, Bergisch Gladbach, Germany). Purity of CD3^+^ cells was 93% to 97% according to flow cytometry analysis. Affymetrix GeneChip microRNA 2.0 Array was used for detection of miRNA expression in the six samples.

### Quantitative RT-PCR analysis

CD3^+^CD45RA^+^CD45RO^-^ naïve T cells and CD3^+^CD45RA^-^CD45RO^+^ non-naïve T cells were isolated from BMMCs by negative selection with immunomagnetic beads. Total RNA was extracted and converted into cDNA, as described in supplemental data. The primer sequences of KLF4, LEF1, SPI1, NR4A2, SIRT1, CDK6, DGKζ and β-ACTIN were shown in Supplementary Table S2.

### Fluorescence-activated cell sorter analysis

The details about cell staining with fluorescence-labeled monoclonal antibodies (mAbs) were described in supplemental data.

### Lentivirus transfection

The recombinant lentivirus LV3-miR34a inhibitor and LV3-NC were purchased from GenePharma (Shanghai, China). The BMMCs obtained from AA patients were cultured in Dulbecco's modified eagle medium (DMEM) supplemented with 10% fetal bovine serum (FBS) and were transfected with LV3-miR34a inhibitor and LV3-NC plus 5 μg/mL polybrene, 1 μg/mL of anti-CD3 mAb, 0.5 μg/mL of anti-CD28 mAb, and 50 U/mL of recombinant human IL-2. Cells with GFP were observed under fluorescence microscope and collected 5 days after transfection.

### T cell activation and proliferation assays

Lymph node cells and splenocytes from miR34a^−/−^ and wild-type mice were left unstimulated or were stimulated with anti-CD3ε and anti-CD28 mAbs (in DMEM, 10% FBS; 18 h). A part of LN cells and splenocytes were used to analyze the T cell activation markers CD69 and CD25 by flow cytometry, while the left were collected to measure the mRNA level of DGKζ. LN cells from nine mice were collected with the same treatment as above to examine the protein level of DGKζ. For proliferation assays, 5-Bromo-2-deoxyuridine (Brdu) and carboxyfluorescein diacetate succinimidyl ester (CFSE) were added to the cell culture, as described in the supplemental data.

### Immunoblot analysis

DGKζ protein level in SAA patients and ERK activation after TCR engagement in LN cells from miR34a^−/−^ mice and wild-type mice were determined by immunoblot. The method was described in detail in the supplemental data.

### Induction of a murine model of bone marrow failure

Recipient CB6F1 mice were pre-irradiated with 5 Gy total body irradiation. Four to six hours later, mice were injected with MHC-mismatched LN (5 × 10^6^ wild-type or miR34a^−/−^) cells through the lateral tail vein [38, 39]. Control mice received 5 Gy TBI only. All mice were bled from the angular vein on day 0 (LN cell infusion day), 3, 7, and 12 as described by Tang *et al*.[38]. Peripheral blood cell counts were performed to observe whether pancytopenia had developed. Treated mice received normal care and nutrition until they died or were euthanized on day 12 to collect tissues for analyses.

### Statistics

All analyses of frequencies, proliferations, cell counts, and mRNA expression for statistically significant differences between two groups were determined by 2-tailed Student's *t*-tests. Comparisons among three groups were performed using one-way ANOVA, followed by the least significant difference (LSD) test. Differences between LV3-miR34a inhibitor and LV3-NC transfection groups were determined by paired sample *t-test*. The correlation analyses were assessed by Spearman's test. Results were presented as mean ± SD. *P* values less than 0.05 were considered statistically significant. All statistical analyses were performed with SPSS18 software.

## SUPPLEMENTARY MATERIALS FIGURES AND TABLES





## Abbreviations

DGK: diacylglycerol kinase
AA: aplastic anemia
BM: bone marrow
BMMCs: bone marrow mononuclear cells
miRNAs: microRNAs
RT-PCR: real time-polymerase chain reaction
DAG: diacylglycerol
Ras-ERK: Ras-extracellular signal regulated kinase
TCR: T cell receptor
LEF1: lymphoid enhancer binding factor 1
SPI1: SPI-1 proto-oncogene
NR4A2: nuclear receptor subfamily 4 group A member 2
SIRT1: sirtuin 1
CDK6: cyclin-dependent kinase 6
inhibitor: lentivirus carrying miR34a inhibitor sequences
LV3-NC: lentivirus carrying non-specific sequences
GFP: green fluorescent protein
LSK: Lin^-^Sca1^+^CD117^+^
LN: lymph node
