# Supplementary material for: Dysregulated miR34a/diacylglycerol kinase ζ interaction enhances T-cell activation in acquired aplastic anemia
Source: Oncotarget. 2016 Dec 20;8(4):6142–54. doi: 10.18632/oncotarget.14046 (PMC5351619; doi:10.18632/oncotarget.14046)
Supplement: Supplementary file 1 [file oncotarget-08-6142-s001.pdf]

Dysregulated miR34a/diacylglycerol kinase ζ interaction enhances T-cell activation in acquired aplastic anemia

Supplementary Materials

SUPPLEMENTARY METHODS

Quantitative RT-PCR analysis

$CD3^+CD45RA^+CD45RO^-$ naïve T cells and $CD3^+CD45RA^-CD45RO^+$ non-naïve T cells were isolated from bone marrow mononuclear cells (BMMCs) by negative selection with immunomagnetic beads. In brief, BMMCs were incubated with a group of microbeads-conjugated antibodies, including anti-CD14, anti-CD16, anti-CD56, anti-CD19, anti-CD15, anti-CD123 and CD235a, together with anti-CD45RA or anti-CD45RO (Miltenyi). The purity of isolated $CD3^+CD45RA^+CD45RO^-$ and $CD3^+CD45RA^-CD45RO^+$ T cells was generally 87% as determined by fluorescence-activated cell sorter analysis. Total RNAs were extracted from the BMMCs of 41 patients and 20 healthy controls as well as naïve T cells and non-naïve T cells from 8 patients and 8 controls with Trizol reagent (Takara, Otsu, Japan). cDNA was prepared using a TaqMan microRNA reverse transcription kit (ABI, Foster City, USA) and a Prime Script reverse transcription reagent kit (Takara). TaqMan real-time RT-PCR was performed to confirm the expression level of miRNAs using TaqMan universal PCR master mix (ABI); U6 served as an internal control. The thermal cycling included 2 minutes at 50°C, 10 minutes at 95°C, then 40 cycles at 95°C for 15 seconds and 60°C for 1 minute.

In order to verify the target genes that play a role in AA, the mRNA levels of Kruppel-like factor 4 (KLF4), lymphoid enhancer binding factor 1 (LEF1), SPI-1 proto-oncogene (SPI1), nuclear receptor subfamily 4 group A member 2 (NR4A2), sirtuin 1 (SIRT1), cyclin-dependent kinase 6 (CDK6), and DGKζ in BMMCs from patients and controls were measured using the SYBR Green RT-PCR Master Mix (Toyobo, Osaka, Japan). All these mRNA data were normalized to the beta-actin mRNA. The level of DGKζ mRNA in mice was examined by the same way. The thermal cycling conditions included an initial denaturation step at 95°C for 30s, followed by 40 cycles of 95°C for 5s, 62°C for 10s, and 72°C for 15s.

All reactions were performed in the light Cycler 480 II (Roche, Rotkreuz, Switzerland). Each sample was examined in triplicate. Relative transcripts were determined by the formula: $2^{-(CTtarget-CTcontrol)}$.

Fluorescence-activated cell sorter analysis

Cells were stained with fluorescence-labeled monoclonal antibodies (mAbs) in 100 μL cell suspensions, incubated for 30 minutes, washed, resuspended in PBS, and subsequently analyzed on a flow cytometer (Beckman Coulter, CA, USA) with Kaluza analysis software. mAbs used in mice targeted CD4, CD8, CD69, CD25, stem cell antigen 1 (Sca1), CD117 (c-kit), and the lineage cocktail (CD3, Gr-1, CD11b, CD45R and Ter-119). mAbs used for human samples targeted CD4, CD8, CD69, and CD25. Each mAb was conjugated with fluorescein isothiocyanate (FITC), phycoerythrin (PE), allophycocyanin (APC), PE-Cy5, PE-Cy7, or peridinin chlorophyll protein (Percp). All mAbs were purchased from BioLegend (San Diego, CA, USA).

T cell proliferation assays

LN cells and splenocytes were similarly stimulated as above mentioned for 72 h, and during the last 12 h were pulsed with 5-Bromo-2-deoxyuridine (Brdu, BD, San Jose, USA). To track cell division, cells were labeled with carboxyfluorescein diacetate succinimidyl ester (CFSE) prior to stimulation. Brdu incorporation and progression of cell division were analyzed by flow cytometry after staining with anti-CD4 and anti-CD8 fluorescence-labeled mAbs.

Immunoblot analysis

To determine DGKζ expression, BMMC were extracted from 8 SAA patients and 8 healthy controls, and lymph node cells from $miR34a^{-/-}$ mice and wild type mice were treated as method Part 'T cell activation' described. To analyze ERK activation after TCR engagement, lymph node cells from 9 $miR34a^{-/-}$ mice and 9 wild type mice were left unstimulated or were stimulated with anti-CD3ε and anti-CD28 mAbs for 5 and 15 min. Human BMMCs and mouse LN cells were lysed using a total protein extraction kit (BestBio, Shanghai, China). Proteins were resolved by SDS-PAGE, transferred to a trans-blot nitrocellulose membrane and probed with antibodies specific to DGKζ (Santa Cruz Biotechnology, Dallas, USA) and phosphorylated ERK1/2 (Cell Signal Technology, Danvers, MA, USA). β-actin or total ERK were determined as loading control.

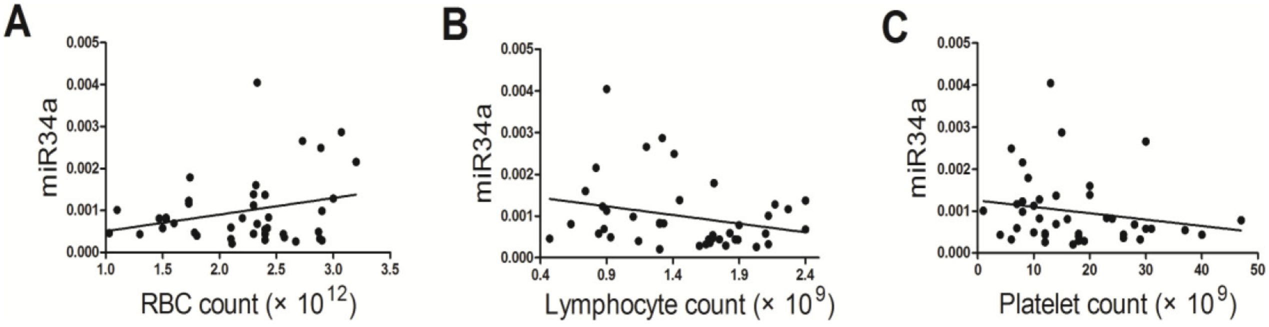

Supplementary Figure S1: No significant correlation was observed between miR34a level and peripheral red blood cell (RBC) count (A), lymphocyte count (B) or platelet count (C) (*P* > 0.05; Spearman's test).

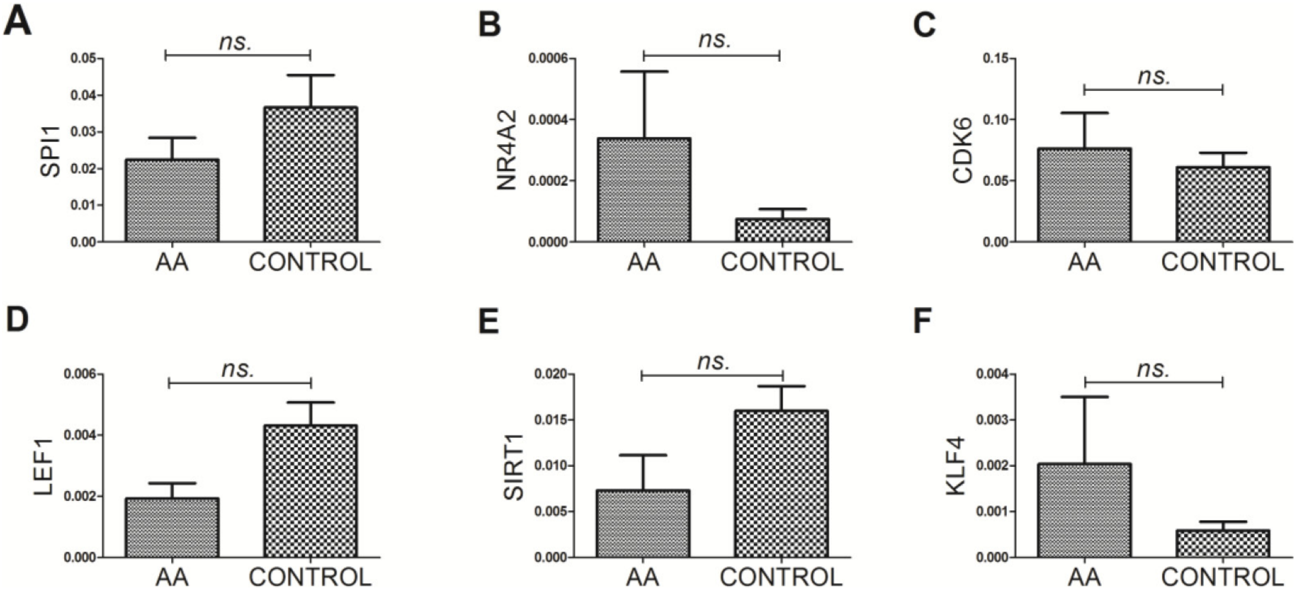

supplementary Figure S2: mRNA level of SPI1, NR4A2, CDK6, LEF1, SIRT1 and KLF4 in AA patients and healthy controls. There was no difference between AA group and control group. Mean ± SEM. *ns*. *P* > 0.05.

Supplementary Table S1: Patients and healthy controls demographics. See Supplementary_Table_S1

Supplementary Table S2: Gene-specific primers for quantitative RT-PCR

Gene	Forward primers	Reverse primers
KLF4	CGGACATCAACGACGTGAG	GACGCCTTCAGCACGAACT
LEF1	TGCCAAATATGAATAACGACCCA	GAGAAAAGTGCTCGTCACTGT
SPI1	GCGACCATTACTGGGACTTCC	GGGTATCGAGGACGTGCAT
NR4A2	GCACTCCGGGTCGGTTTAC	GCCACGTAGTTCTGGTGGAA
SIRT1	TAGCCTTGTCAGATAAGGAAGGA	ACAGCTTCACAGTCAACTTTGT
CDK6	TCTTCATTCACACCGAGTAGTGC	TGAGGTTAGAGCCATCTGGAAA
DGKζ	CCGCTTTCGGAATAAGATGTTCT	AACAACACACTGGGGTTTCAG
β-ACTIN	GAAGTGTGACGTGGCATCC	CCGATCCACACGGAGTACTT
mDgkζ	CTGAGGAGCAGATCCAGAGC	TCCCCGACATAGCAGAAGTC
mβ-actin	GGCTGTATTCCCCTCCATCG	CCAGTTGGTAACAATGCCATGT

First 8 sequences are human primers; mDgkζ and mβ-actin are murine primers

Supplementary Table S3: Pancytopenia induced by miR34a$^{-/-}$ and wild-type LN cells on day 12

Treatment	*n*	WBC (×10^{9}/L)	RBC (×10^{12}/L)	PLT (×10^{9}/L)
TBI only	8	0.80 ± 0.10	7.67 ± 1.53	653 ± 228
TBI + WT LN	12	0.20 ± 0.07	4.84 ± 0.61	86 ± 46
TBI + miR34a$^{-/-}$ LN	12	0.22 ± 0.11	5.28 ± 1.28	132 ± 77
Statistics		*$P > 0.05$	*$P > 0.05$	*$P > 0.05$

Means ± SD after variance analysis. WBC, white blood cells; RBC, red blood cells; PLT, platelets; TBI, total body irradiation; LN, lymph node; WT, wild-type. *TBI + WT LN *vs* TBI + miR34a$^{-/-}$ LN
