# Supplementary material for: Dysregulated miR34a/diacylglycerol kinase ζ interaction enhances T-cell activation in acquired aplastic anemia
Source: Oncotarget. 2016 Dec 20;8(4):6142–54. doi: 10.18632/oncotarget.14046 (PMC5351619; doi:10.18632/oncotarget.14046)
Supplement: Supplementary file 2 [file oncotarget-08-6142-s002.docx]

**Supplementary Table S1. Patients and healthy controls demographics**

| **Patient no.** | **Sex** | **Age, y** | **NEU (×10^9^/L)** | **PLT(×10^9^/L)** | **RET (×10^9^/L)** | **Severity** |
| --- | --- | --- | --- | --- | --- | --- |
| 1 | F | 37 | 0.33 | 4 | 0 | SAA |
| 2 | M | 69 | 0.3 | 14 | 20 | SAA |
| 3 | F | 31 | 1.4 | 26 | 60 | MAA |
| 4 | M | 33 | 0.67 | 12 | 30 | MAA |
| 5 | M | 43 | 0.15 | 30 | 10 | SAA |
| 6 | F | 29 | 0.28 | 10 | 40 | SAA |
| 7 | M | 40 | 0.78 | 1 | 10 | SAA |
| 8 | M | 15 | 0.03 | 20 | 10 | SAA |
| 9 | M | 50 | 0.28 | 18 | 10 | SAA |
| 10 | F | 39 | 0.31 | 10 | 10 | SAA |
| 11 | F | 56 | 0.78 | 11 | 10 | SAA |
| 12 | F | 33 | 0.99 | 29 | 70 | MAA |
| 13 | M | 56 | 0.12 | 15 | 20 | SAA |
| 14 | F | 22 | 1.77 | 17 | 30 | MAA |
| 15 | M | 20 | 0.15 | 14 | 10 | SAA |
| 16 | F | 43 | 0.23 | 23 | 10 | SAA |
| 17 | M | 16 | 0.31 | 28 | 30 | MAA |
| 18 | M | 23 | 0.34 | 20 | 10 | SAA |
| 19 | F | 58 | 0.06 | 13 | 0 | SAA |
| 20 | M | 27 | 0.7 | 31 | 60 | MAA |
| 21 | M | 63 | 1.07 | 12 | 40 | MAA |
| 22 | F | 27 | 0.02 | 8 | 0 | SAA |
| 23 | F | 57 | 0.51 | 40 | 80 | MAA |
| 24 | M | 33 | 0.23 | 9 | 20 | SAA |
| 25 | F | 56 | 0.52 | 7 | 20 | MAA |
| 26 | M | 22 | 0.43 | 37 | 50 | MAA |
| 27 | M | 47 | 0.12 | 6 | 0 | SAA |
| 28 | F | 64 | 0.7 | 19 | 50 | MAA |
| 29 | M | 77 | 0.35 | 6 | 30 | SAA |
| 30 | M | 59 | 0.18 | 16 | 0 | SAA |
| 31 | M | 35 | 0.04 | 8 | 0 | SAA |
| 32 | F | 38 | 0.34 | 18 | 10 | SAA |
| 33 | F | 44 | 1.28 | 11 | 40 | MAA |
| 34 | F | 40 | 0.78 | 24 | 10 | MAA |
| 35 | M | 51 | 0.31 | 26 | 10 | SAA |
| 36 | M | 59 | 1.05 | 18 | 40 | MAA |
| 37 | F | 42 | 0.94 | 12 | 10 | SAA |
| 38 | F | 57 | 0.68 | 7 | 50 | MAA |
| 39 | M | 38 | 0.2 | 30 | 10 | SAA |
| 40 | F | 20 | 1.23 | 47 | 20 | MAA |
| 41 | F | 48 | 0.21 | 8 | 20 | SAA |
| 42 | F | 43 | 0.39 | 23 | 10 | SAA |
| 43 | F | 26 | 0.03 | 19 | 10 | SAA |
| 44 | M | 54 | 0.07 | 23 | 0 | SAA |
| 45 | M | 21 | 0.33 | 37 | 10 | SAA |
| 46 | F | 33 | 0.6 | 9 | 10 | SAA |
| 47 | M | 47 | 1.2 | 12 | 0 | SAA |
| 48 | M | 32 | 1.03 | 10 | 10 | SAA |
| 49 | F | 45 | 0.06 | 18 | 30 | SAA |
| 50 | F | 27 | 4.7 | 170 | 80 | Healthy |
| 51 | M | 44 | 4.6 | 123 | 90 | Healthy |
| 52 | M | 36 | 4.34 | 142 | 70 | Healthy |
| 53 | F | 45 | 2.12 | 146 | 50 | Healthy |
| 54 | F | 21 | 3.88 | 165 | 80 | Healthy |
| 55 | F | 28 | 3.21 | 157 | 60 | Healthy |
| 56 | M | 33 | 2.6 | 134 | 90 | Healthy |
| 57 | M | 46 | 2.44 | 143 | 90 | Healthy |
| 58 | F | 32 | 2.33 | 120 | 80 | Healthy |
| 59 | F | 23 | 3.1 | 133 | 80 | Healthy |
| 60 | M | 26 | 5.43 | 150 | 70 | Healthy |
| 61 | F | 33 | 3.82 | 183 | 60 | Healthy |
| 62 | F | 45 | 4.89 | 163 | 40 | Healthy |
| 63 | M | 35 | 3.3 | 167 | 50 | Healthy |
| 64 | M | 47 | 4.46 | 210 | 80 | Healthy |
| 65 | F | 37 | 6.2 | 177 | 90 | Healthy |
| 66 | M | 28 | 3.8 | 268 | 80 | Healthy |
| 67 | M | 56 | 4.34 | 144 | 90 | Healthy |
| 68 | F | 41 | 6.59 | 181 | 60 | Healthy |
| 69 | F | 22 | 4.87 | 264 | 40 | Healthy |
| 70 | F | 60 | 5.73 | 189 | 50 | Healthy |
| 71 | M | 63 | 6.92 | 264 | 70 | Healthy |
| 72 | M | 44 | 3.24 | 148 | 80 | Healthy |
| 73 | F | 55 | 5.78 | 122 | 50 | Healthy |
| 74 | F | 40 | 4.77 | 218 | 40 | Healthy |
| 75 | M | 33 | 2.53 | 136 | 80 | Healthy |
| 76 | M | 45 | 3.49 | 142 | 60 | Healthy |
| 77 | M | 29 | 5.61 | 230 | 80 | Healthy |

NEU indicates neutrophil; PLT, platelet; RET, reticulocyte; y, year; M, male; F, female; SAA, severe aplastic anemia; MAA, moderate aplastic anemia
